# Impaired inactivation of the G protein‐coupled receptor Kinase 2 contributes to tau pathogenesis in human AD Neurons

**DOI:** 10.1002/alz.095110

**Published:** 2025-01-09

**Authors:** Thais Rafael Guimaraes, Matthew L MacDonald, Amantha Thathiah

**Affiliations:** ^1^ University of Pittsburgh, Pittsburgh, PA USA

## Abstract

**Background:**

Aggregation of the hyperphosphorylated tau protein in neurofibrillary tangles (NFTs) is a pathological hallmark of Alzheimer’s Disease (AD). Several kinases contribute to the pathological phosphorylation of tau (pTau). However, kinase‐targeted therapies for AD have failed in clinical trials due to largely unknown mechanism of action, low efficacy, and severe side‐effects. G protein‐coupled receptor (GPCR) kinases (GRKs) have been implicated in neurological disorders, such as Parkinson’s Disease, through phosphorylation of non‐GPCR substrates, e.g., α‐synuclein. We previously showed that GRK2 is abundantly expressed in neurons, positively correlates with soluble tau levels, and is associated with NFTs in the human AD brain. However, whether GRK2 is causally involved in tau pathobiology remains unknown.

**Method:**

We utilized a comprehensive toolbox of molecular, cellular, and biochemical techniques to investigate the impact of GRK2 on tau phosphorylation in CRISPR/Cas9‐modified HEK293cell lines, patient‐derived induced neurons (iNs), and post‐mortem human AD brain tissue.

**Result:**

Using unbiased mass spectrometry analysis, we first show that GRK2 overexpression induces global changes in the tau phosphoproteome, specifically increasing levels of AD‐associated pTau sites (e.g., T181, T217, T231, and S396/404). We further show that GRK2 directly interacts with tau and pTau species and, notably, that this interaction is enhanced in AD patient‐derived iNs. Previous studies have shown that ERK inactivates GRK2 via phosphorylation at residue S670 and could provide a putative insight into GRK2 dysfunction in AD. Strikingly, we find that inactive GRK2‐S670 levels are decreased in AD patient brains, suggesting that impaired GRK2 inactivation contributes to the pTau burden observed in AD patients. Accordingly, we show that the ERK‐mediated inactive form of GRK2 (GRK2‐S670D) protects against elevated pTau levels. Furthermore, pharmacological inhibition of GRK2 in human iNs decreases pTau levels and specifically increases both ERK activation and GRK2‐S670 levels in AD‐derived iNs. Lastly, GRK2 inhibition reduces the oxidative stress observed in AD iNs, suggesting that GRK2 inactivation can suppress disease‐associated neurotoxicity.

**Conclusion:**

These studies elucidate direct mechanisms via which GRK2 mediates tau phosphorylation in AD neurons. Further investigation of GRK2‐mediated therapeutic intervention strategies may reveal novel avenues to modulate tau pathogenesis and consequent neurodegeneration in AD.

